# Associations between Serum Vitamin A and Metabolic Risk Factors among Eastern Chinese Children and Adolescents

**DOI:** 10.3390/nu14030610

**Published:** 2022-01-30

**Authors:** Ting Tian, Yuanyuan Wang, Wei Xie, Jingxian Zhang, Yunlong Ni, Xianzhen Peng, Guiju Sun, Yue Dai, Yonglin Zhou

**Affiliations:** 1 Institute of Food Safety and Assessment, Jiangsu Provincial Center for Disease Control and Prevention, Nanjing 210009, China; jstt@jscdc.cn (T.T.); wyypro@foxmail.com (Y.W.); jscdcxiewei@sina.com (W.X.); z18252063009@163.com (J.Z.); nyl2008@163.com (Y.N.); 18915999341@163.com (Y.D.); 2 Department of Nutrition and Food Hygiene, School of Public Health, Southeast University, Nanjing 210009, China; gjsun@seu.edu.cn; 3 Department of Public Health and Preventive Medicine, Kangda College of Nanjing Medical University, Lianyungang 222000, China; xianzhenpeng@njmu.edu.cn

**Keywords:** vitamin A, metabolic syndrome, children and adolescents, metabolic risk factors

## Abstract

Vitamin A, a fat-soluble essential vitamin, is implicated in a large range of physiological processes. Up to now, the associations between vitamin A and metabolic syndrome (MetS) or other metabolic risk factors are controversial in children and adolescents. Thus, we aimed to dig into the relationship of vitamin A with MetS and many other metabolic risk factors. This was a cross-sectional study derived from the China National Nutrition and Health Surveillance of Children and Lactating Mothers. A total of 3025 school-aged (7–17 years) children and adolescents were selected by applying multistage stratified cluster random sampling methods in the Jiangsu Province of eastern China. Through enquiry survey, anthropometric measurement and laboratory examination, relevant information and blood biochemical indexes of the participants were collected in this study. MetS was identified according to the modified criteria of the National Cholesterol Education Program–Adult Treatment Panel III (NCEP-ATP III). Multivariate logistic analysis and the generalized additive model (GAM) were used to analyze the relationship between vitamin A and various metabolic risk factors. The overweight, obesity and MetS prevalence of children and adolescents in this study was 14.0%, 11.9% and 5.1%, respectively. The risk of prevalent MetS, general obesity, high low-density lipoprotein (LDL), high total cholesterol (TC) and hyperuricemia increased with vitamin A in a dose-dependent way. Logistic regression analysis showed that serum vitamin A Z scores were positively associated with MetS and central obesity, elevated blood pressure (BP) and elevated triglyceride (TG). Sex stratification analysis showed that both in male and female participants, the risk of prevalent MetS, general obesity, high LDL, high TC and hyperuricemia still increased with vitamin A levels. MetS was at a high prevalence level in children and adolescents in Jiangsu that were 7–17 years old. Vitamin A was positively associated with obesity, MetS, dyslipidemia and hyperuricemia. More public health measures and new visions should focus on the effects of retinol on children and adolescents.

## 1. Introduction

Vitamin A, namely retinol and its derivatives, is the key nutrient implicated in a myriad of physiological processes, such as maintaining the function of the immune system, normal vision, growth and metabolisms in human beings [1,2,3]. It is generally established that vitamin A is a fat-soluble essential vitamin which cannot be synthetized in the human body and only can be ingested from exogenous plants or animal foods [4]. Normally, chronic low intake of vitamin A from diets can cause vitamin A deficiency (VAD) and then result in night blindness, impaired immune function and even increased child mortality [5,6].

However, excessive intake of vitamin A can lead to both acute and chronic toxicity effects, which are well documented in many researches. The preformed vitamin A intakes are only twice the current recommended dietary allowance (RDA) in association with osteoporosis and hip fracture [7].

The most common cause of mortality among adults around the world is cardiovascular diseases which root in childhood, underscoring the necessity to identify and intervene metabolic risk factors in children and adolescents [8]. In 2015, a total of 107.7 million children were obese globally. Strong evidence from Chinese studies has estimated the prevalence of obesity was 7.9% in 6–17 years for 2015–19 [9]. Metabolic syndrome (MetS) is the clustering of central obesity, elevated blood pressure, elevated fasting blood glucose (FBG), low high-density lipoprotein (HDL) and high triglycerides (TG) [10]. It has been widely recognized that children who are at risk of developing the MetS and other metabolic risk factors, including dyslipidemia and hyperuricemia, may lead to type 2 diabetes mellitus and cardiovascular disease in later life [11,12].

So far, the association between vitamin A and MetS or other metabolic risk factors are controversial in children and adolescents. American national health and nutrition examination surveys (NHANES) data showed vitamin A had a positive relationship with HOMA-IR, uric acid and MetS [13]. Nevertheless, one study had inverse results that vitamin A level was negatively associated with hypertriglyceridemia, obesity and MetS among Chinese Chongqing school-aged children [14]. Furthermore, a study indicated that during the weight loss program, vitamin A plasma levels increased significantly in 12–17 year old adolescents [15]. These studies make the relationship between vitamin A and metabolic risk factors extremely confusing. Besides, it is worth noting that many of these studies have some limitations, for instance, in a non-representative population, or with a limited sample size, or with a short age span, or comparing less metabolic risk factors.

Thus, this study capitalized on a provincial representative larger sample through multistage stratified cluster random sampling methods in Chinese children and adolescents and attempted to explore the association of serum vitamin A with MetS and many other metabolic risk factors.

## 2. Methods

### 2.1. Study Participants

This was a cross-sectional study derived from the China National Nutrition and Health Surveillance of Children and Lactating Mothers, which was partially conducted in Jiangsu Province, located in the east of China, in 2016–2017. A multistage stratified cluster random sampling method was applied to select representative study participants. First, 12 survey sites were randomly selected from the whole province, including 2 sites in large cities, 8 sites in medium cities and 2 sites in normal rural countries. Then, two towns/subdistricts at each survey site were randomly picked. Third, two communities or villages of towns/subdistricts were selected. Last, 70 participants were randomly picked. At each site, 280 subjects aged 7–17 years living in the area more than 12 months before the survey and volunteering to participate in enquiry survey, anthropometric measurement and blood drawing, were chosen. A total of 3360 school-aged children and adolescents were investigated. After excluding those who had acute infectious diseases, autoimmune diseases or other serious diseases which were not suitable for the survey and the lack of key data, such as demographic information, lab testing information, or anthropometric information (*n* = 335), 3025 participants were enrolled into the final analysis. This study was carried out in accordance with the Declaration of Helsinki and approved by the ethical committee of the China Center for Disease Control and Prevention. The ethical approval number was 201614. All the participants provided signed informed content and agreed to attend this survey.

### 2.2. Survey and Measurements

Enquiry Survey: Face to face interviews were conducted by the unified trained investigators with the structured questionnaires provided by the national project group. The information of age, sex, physical activity and screen time were collected in the interview. All the disease conditions of the participants were referred to their medical diagnosis results. Screen time refers to the time of using the electronic screen every day. The short time is <2 h, and the long time is ≥2 h. If the exercise activity reaches the above exercise level of 0–3 days a week, it is defined as a low physical activity level. Providing that the exercise level reaches above 4 days a week, it is defined as a high physical activity level.

Anthropometric measurement: After the interview, the investigator measured the height, weight, waist circumference (WC), systolic blood pressure (SBP) and diastolic blood pressure (DBP) of the participants. These measurements strictly followed the standard protocol and were conducted under strict quality control. Height and weight were measured without shoes and coats. WC was measured by a soft ruler in the fasting states, and the measure location was at the middle point between the bottom of the rib cage and the uppermost border of the iliac crests at the end of exhalation. The standard mercury sphygmomanometer was used to take BP readings from participants after 5 min of quiet sitting. The mean of three measures of BP was recorded for further analysis.

Laboratory examination: 6 mL morning fasting blood was taken from each participant. Vitamin A was tested by high-performance liquid chromatography (HPLC), according to standard method recorded in the National Health Standard (method for vitamin A deficiency screening) from the National Health Commission of the People’s Republic of China [16]. The serum was added with internal standard retinol acetate, extracted with n-hexane, separated by HPLC C18 reversed-phase column and quantitatively detected by UV detector. Other blood biochemical indexes, such as uric acid, were measured using colorimetry (Roche C702 automagical analyzer). Triglyceride (TG), total cholesterol (TC), low-density lipoprotein cholesterol (LDL-C) and high-density lipoprotein cholesterol (HDL-C) were tested using enzyme colorimetry (Roche Cobas C701 automatic biochemical analyzer series), and fasting blood glucose (FBG) was measured using glucokinase method (Roche P800 automatic biochemical analyzer).

### 2.3. Diagnostic Criteria and Definitions

Overweight and obesity were defined by the gender- and age-specific BMI cutoffs for Chinese children and adolescents [17]. The geographical region was dichotomized into rural and urban areas.

MetS were identified according to the modified criteria of the National Cholesterol Education Program–Adult Treatment Panel III (NCEP-ATP III) [18,19]. At least three of the five components are as follows:

(1) central obesity: WC ≥ age- and gender-specific 90th percentile [20]; (2) elevated TG: TG ≥ 1.24 mmol/L; (3) low HDL: HDL ≤ 1.03 mmol/L; (4) elevated blood pressure: SBP and/or DBP ≥ 90th percentile for gender, age and height [21]; (5) elevated FBG: glucose ≥ 6.1 mmol/L.

Elevated TC: TC ≥ 5.18 mmol/L [22]; elevated LDL: LDL ≥ 3.36 mmol/L [22].

Hyperuricemia: serum uric acid (SUA) ≥ 357μmol/L [23].

Obesity phenotype: Participants were classified into four metabolic phenotypes according to the obesity and metabolic syndrome status. (1) metabolically healthy non-obese (MHNO); (2) obese but absence of MetS (MHO); (3) non-obese subjects with MetS (MNHNO); (4) obese subjects with MetS (MNHO) [24].

### 2.4. Statistical Analysis

The statistical analysis was performed on the R software (Version 4.1.0). Characteristics of the participants were presented as counts (proportions) or median (interquartile range, IQR). Serum vitamin A level was divided into quartiles, quartile 1 (Q1) was <0.323 mg/L, Q2 was [0.323–0.377) mg/L, Q3 was [0.377–0.430) mg/L and Q4 was ≥0.430 mg/L. Variables were compared using the Variance test, Kruskal–Wallis test or Pearson’s chi-square test between vitamin A quartiles when appropriate. Multivariate logistic analysis was used to analyze the relationship between vitamin A quartiles and various metabolic risk factors. Adjusted Odds ratios (ORs) with a 95% confidence interval (CI) were presented after adjusting age, gender, screen time and physical activity time. Tests for linear trends were performed by entering the continuous variable directly in the multivariate logistic analysis. A generalized additive model (GAM) was used to visualize the nonlinear associations between serum vitamin A and selected metabolic risk factors. Additionally, the serum vitamin A concentrations were converted to a Z-score by this equation: (vitamin A − vitamin A_mean_)/vitamin A_SD_. We performed the multivariate logistic analysis to evaluate the association of MetS and its components with vitamin A Z-scores. The *p*-value < 0.05 was considered statistically significant, and all statistical hypothesis tests were two-sided.

## 3. Results

### 3.1. Characteristics of Participants

This study enrolled 3025 participants aged from 7–17 years. Among these participants, 1520 (50.2%) were males, and 1505 (49.8%) were females. The median (interquartile range, IQR) concentration of serum vitamin A of all study participants was 0.377 mg/L (0.323–0.430 mg/L). Vitamin A levels were not significantly different between males and females (*p* = 0.962). Characteristics of participants are displayed in Table 1 across quartiles of serum vitamin A concentrations. Age, residence, anthropometric indexes and biochemistry indexes were remarkably different among vitamin A quartiles (all *p* < 0.05). Besides, linear trends of the above variables were found across vitamin A quantiles (all *p* for trend < 0.05). However, there were no differences or linear trends in physical activity and screen time of the children and adolescents in this study.

### 3.2. Prevalence of Metabolic Diseases across Serum Vitamin A Quartiles in Children and Adolescents

The overweight and obesity prevalence of children and adolescents in this study was 14.0% and 11.9%. The overall prevalence of MetS was 5.1% for all participants. As to MetS components, 18.7% of all participants had abdominal obesity, 41.3% had elevated BP, 3.5% had elevated FBG, 4.1% had low HDL and 15.0% had high TG, respectively. Table 2 present the prevalence of metabolic phenotypes of obesity according to vitamin A quartiles. Participants with increased vitamin A levels were more prevalent in overweight and obesity (*p* for trend < 0.001). From the Q1 to Q4 level, the prevalence of overweight was increasing from 9.4% to 17.9%, and the prevalence of obesity was rising from 7.7% to 18.7%. With the increase of serum vitamin A quartiles, MetS became more prevalent (*p* for trend < 0.001). There was a significant difference in obesity phenotypes among vitamin A quartiles (*p* < 0.001). Proportions of MHNO were decreased, and MHO, MNHNO and MNHO were more prevalent in higher vitamin A quartiles.

### 3.3. Associations of Serum Vitamin A with Metabolic Risk Factors in Children and Adolescents

**Figure 1:**
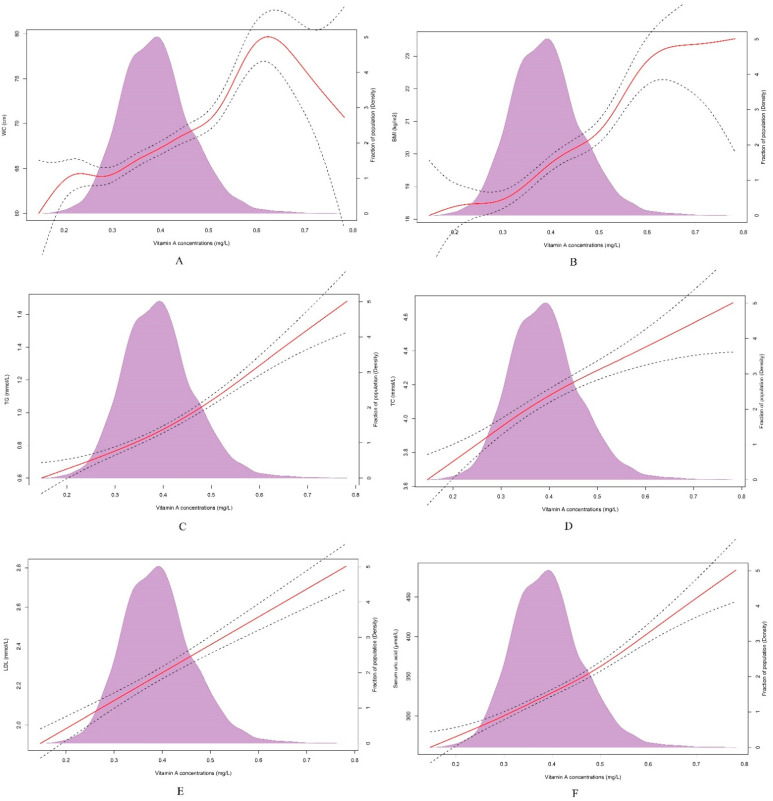
Dose-response curves with 95% confidence interval (CI) were modelled for the associations of serum vitamin A and numerous metabolic risk factors by the generalized additive model (GAM). (**A**). the association between vitamin A concentrations and WC. (**B**). the association between vitamin A concentrations and BMI. (**C**). the association between vitamin A concentrations and TG. (**D**). the association between vitamin A concentrations and TC. (**E**). the association between vitamin A concentrations and LDL. (**F**). the association between vitamin A concentrations and SUA. Abbreviations: waist circumference (WC), body mass index (BMI), triglyceride (TG), total cholesterol (TC), low-density lipoprotein cholesterol (LDL), serum uric acid (SUA).

**Figure 2:**
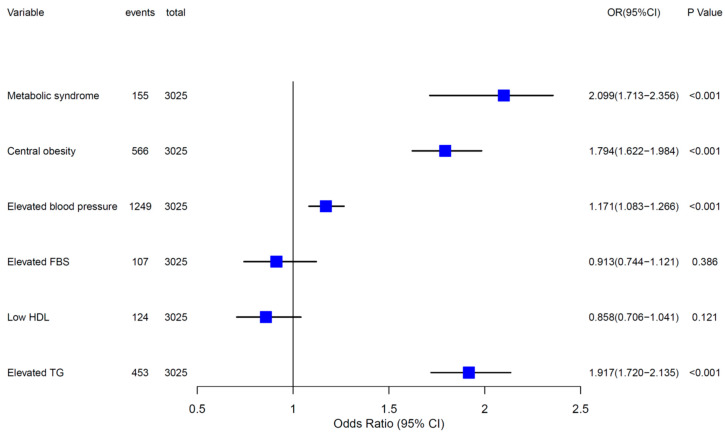
The forest plot of logistic regression analysis results for the associations of serum vitamin A Z scores with MetS and its components. Vitamin A was converted to a z-score by this equation: (Vitamin A − Vitamin A_mean_)/Vitamin A_SD_.

In this study, we found that pronounced relationships existed between vitamin A levels and various metabolic risk factors. In Table 3, compared to the Q1 vitamin A level as a reference, Q2, Q3 and Q4 level participants were in greater danger of general obesity, high LDL, high TC and hyperuricemia, and all the adjusted ORs were more than one. With the increase of vitamin A quartiles, the risk of having MetS and parts of its components, such as central obesity, elevated BP and high TG, also raised (*p* for trend < 0.001). Compared to the Q1 reference vitamin A quartile, the adjusted odds ratios (ORs) for MetS of the Q3 and Q4 quantiles were 2.244 (95% CI: 1.221–4.123) and 5.257 (95% CI: 2.968–9.310). We further adopted the GAM to visualize the nonlinear associations between serum vitamin A and selected metabolic risk factors. The positive dose-dependent relationships between serum vitamin A and WC, BMI, TG, TC, LDL and SUA, can be easily understood from Figure 1. In Figure 2, logistic regression analysis showed that serum vitamin A Z scores were positively associated with MetS (OR = 2.009, 95%CI: 1.713–2.356), central obesity (OR = 1.794, 95% CI: 1.622–1.984), elevated BP (OR = 1.171, 95% CI: 1.083–1.266) and elevated TG (OR = 1.917, 95% CI: 1.720–2.315).

Furthermore, we conducted a stratification logistic analysis by sex in Supplementary Tables S1 and S2. Both in male and female participants, the risk of prevalent MetS, general obesity, high LDL, high TC and hyperuricemia still increased with vitamin A quartiles.

## 4. Discussion

So far, the roles of vitamin A in obesity, MetS or other metabolic risk factors among children and adolescents are vague. Thus, we used this large-scale cross-sectional study with a strict sampling method to explore the associations between vitamin A and a variety of metabolic risk factors among Chinese representative 7–17 year old participants. In this study, we calculated the obesity prevalence and the prevalent MetS of selected school-aged participants. Furthermore, we observed intriguing positive associations between serum vitamin A and various metabolic risk factors in our research populations.

The prevalence of overweight and obesity among the Jiangsu children and adolescents of our study was 14.0% and 11.9%, which was consistent with the results from a Chinese national survey in 2015, with 14.0% of overweight and 10.5% of obesity [25]. As a case in point, that indicated the stability and reliability of our results, and we must highlight the attention to prevent and reduce weight in numerous overweight and obese children and adolescents. In addition, MetS was prevalent in 5.1% of participants, higher than the national average of 3.37% in the same criteria [26]. MetS is a group of metabolic disorders at a high prevalence level in the world, becoming a health-threatening public health problem, and these risks increasingly begin in childhood and adolescence [11,27]. We are reminded of the importance and urgency of taking preventive measures against MetS among children and adolescents. Combined obesity and MetS condition, MHO, MNHNO and MNHO were more prevalent in higher vitamin A quartiles. This meant the degree of metabolic disorder also increased with the serum vitamin A levels.

In our study, positive associations were observed between serum vitamin A and a number of metabolic risk factors. We observed that MetS, obesity, high LDL, high TC and hyperuricemia were more prevalent in higher vitamin A level school-aged participants. To be more specific, it turned out that serum vitamin A was significantly connected to prevalent obesity and had a positive dose-response relationship with BMI in children and adolescents. Previous studies in Chinese children and adolescents had demonstrated similar results. The China National Nutrition and Health Survey 2010–2013, a nationally representative cross-sectional study, reported that the average serum retinol concentration was notably higher in overnutrified than non-overnutrified children and adolescents [28]. These were in accordance with some other studies findings [29,30]. However, some studies showed different results, which found that low serum concentrations of retinol were associated with higher BMI [14,31]. The different associations possibly appeared on account of the fact that these studies chose a population with higher BMI as subjects, such as morbidly obese patients, children with high adiposity or less representative subjects. Moreover, the difference in the relationship between serum vitamin A and BMI showed that the effect of retinol on fat biology might be different in various BMI individuals. This can be explained by the fact that vitamin A, as a fat-soluble vitamin, and its active form participate in lipid metabolism, which is relevant to increasing concentrations of blood lipids [32].

When it comes to MetS, among metabolic components, abdominal obesity, high TG levels and elevated blood pressure were positively associated with serum retinol. Analogously, data from the Korea National Health and Nutrition Examination Survey found positive dose-dependent associations between serum retinol and MetS [33]. Moreover, among U.S. adolescents, retinol exhibited a positive relationship with MetS outcomes, data from NHANES 2001–2006 [13]. Nevertheless, these findings were different from some studies. A meta-analysis suggested that no association was detected between retinol and MetS [34]. A cross-sectional study in Chinese Chongqing City found vitamin A insufficiency significantly associated with MetS among urban school-age children, which was disparate from our results [14]. These discrepant findings are unclear, and it might be due to differences in sample representativity (e.g., general population vs. only urban population), gender, age groups, adjusted compounders and study designs. A randomized controlled trial observed that MetS subjects had a trend toward a higher post-prandial response to a physiologically relevant dose of preformed vitamin A and had higher bioavailability as compared to healthy controls [35]. Retinol binding protein 4 (RBP4), forming a 1:1:1 m complex with retinol and thyroxine carrier protein in the blood, an appropriate surrogate marker for serum vitamin A level, was also in line with the increased risk of hypertriglyceridemia, high blood pressure [36,37,38]. On the other hand, several mechanisms regarding the adverse effects of vitamin A and vitamin A-related parameters on metabolic health have been proposed. β-oxidative activity is a crucial pathogenic factor of MetS [39]. Vitamin A may be a vital β-regulator of oxidative activity. Retinoic acid, a derivative of vitamin A, can stimulate mitochondria in human hepatocytes β-oxidation [40]. An animal model showed that the β-oxidation of fatty acid mitochondria in retinoic acid receptor-deficient mice was lower than that of wild-type mice [41].

In addition, we explored the relationship between TC, LDL level and serum vitamin A level. Positive associations were found between TC, LDL level and serum vitamin A level. High TC and high LDL were more common in subjects with higher serum vitamin A levels. Analogous research conclusions were referred to children in Brazil and Poland [42,43]. One metabolomic profile on plasma retinol associated dyslipidemia, revealed that elevated retinol levels might be associated with hormone metabolism and inflammation status. Different lipid metabolic pathways, including glycerophospholipid, glycosylphosphatidylinositol (GPI) and steroid hormone biosynthesis pathways might explain the effects of vitamin A in dyslipidemia [44].

It was indicated that SUA was positively raised with vitamin A levels and hyperuricemia was more prevalent in children and adolescents with higher serum vitamin A levels in our research. The relationship between vitamin A and hyperuricemia was revealed in many studies consisting of both adults and adolescents [13,45,46]. However, so far, the deep level mechanism of this phenomenon has not yet been fully illustrated. One widely accepted explanation is that high serum retinol levels raised along with the increase of xanthine oxidase activity, which plays a critical role in increasing serum uric acid levels. Xanthine oxidase converts retinol to retinoic acid and catalyzes xanthine oxidation to produce uric acid [47].

This study had some limitations. First, this was a cross-sectional design and restricted the ability to access the causality, and further pathogenic mechanisms should be verified by experiments. Second, in view of the liver being the main reservoir of vitamin A, serum retinol levels might not reflect excessive vitamin A states in the human body. Third, we acknowledged that these findings were valid for the Chinese ethnicity, and further confirmation should be obtained in a wide range of populations. Nevertheless, we do have some strengths in this study. Through the scientific sampling method and rigorous quality control, large regional representative participants were included in this research. Furthermore, this study is full of innovations by comparing serum vitamin A levels with numerous metabolic risk factors among Chinese children and adolescents.

## 5. Conclusions

MetS was at a high prevalence level in Jiangsu 7–17-year-old children and adolescents. Vitamin A was positively associated with numerous metabolic risk factors, such as obesity, MetS, dyslipidemia and hyperuricemia. More public health measures and new visions should focus on the effects of retinol on children and adolescents. Furthermore, tailored strategies and more prospective studies should be condcuted to manage vitamin A levels in children and adolescents.

## Figures and Tables

**Table 1:** Basic information of male and female participants.

Variables	Serum Vitamin A Levels				*p*-Value	*p* for Trend
	Q1 (*n* = 756)	Q2 (*n* = 757)	Q3 (*n* = 762)	Q4 (*n* = 750)		
Age, years	10.2 ± 2.7	10.8 ± 2.8	11.8 ± 3.0	12.8 ± 2.7	<0.001	<0.001
Residence					<0.001	<0.001
Urban	596 (78.8)	638 (84.3)	655 (86.0)	642 (85.6)		
Rural	160 (21.2)	119 (15.7)	107 (14.0)	108 (14.4)		
Physical activity					0.114	0.151
Low	460 (60.8)	482 (63.7)	442 (58.0)	442 (58.9)		
High	296 (39.2)	275 (36.3)	320 (42.0)	308 (41.1)		
Screen time					0.708	0.700
Low	657 (86.9)	672 (88.8)	673 (88.3)	658 (87.7)		
High	99 (13.1)	85 (11.2)	89 (11.7)	92 (12.3)		
Anthropometrics						
Height (cm)	141.8 (132.3, 155.2)	146.6 (135.1, 160.2)	155.6 (141.9, 164.4)	160.0 (150.9, 168.0)	<0.001	<0.001
Weight (kg)	34.5 (27.4, 46.0)	39.1 (29.8, 50.6)	47.2 (34.5, 57.2)	52.6 (42.2, 63.1)	<0.001	<0.001
WC (cm)	59.9 (54.3, 65.7)	62.1 (56.4, 69.1)	65.7 (59.0, 73.0)	69.3 (62.9, 77.8)	<0.001	<0.001
BMI (kg/m^2^)	16.8 (15.4, 19.2)	17.8 (15.8, 20.4)	19.1 (16.7, 22.0)	20.5 (17.9, 23.3)	<0.001	<0.001
SBP (mmHg)	111.7 (104.3, 119.3)	114.0 (106.0, 122.3)	115.7 (107.7, 123.7)	117.3 (110.3, 125.7)	<0.001	<0.001
DBP (mmHg)	66.7 (61.3, 73.0)	67.0 (61.7, 73.3)	67.3 (62.3, 72.3)	69.0 (64.7, 74.0)	<0.001	<0.001
Biochemistry						
FBG (mmol/L)	5.2 (4.9, 5.5)	5.2 (4.9, 5.5)	5.3 (5.0, 5.6)	5.3 (5.0, 5.6)	0.010	0.036
TG (mmol/L)	0.7 (0.6, 0.9)	0.8 (0.6, 1.0)	0.8 (0.6, 1.1)	1.0 (0.7, 1.3)	<0.001	<0.001
TC (mmol/L)	3.9 (3.5, 4.3)	4.1 (3.7, 4.6)	4.0 (3.6, 4.5)	4.1 (3.7, 4.7)	<0.001	<0.001
LDL (mmol/L)	2.1 (1.8, 2.4)	2.2 (1.9, 2.6)	2.2 (1.8. 2.6)	2.3 (1.9, 2.7)	<0.001	<0.001
HDL (mmol/L)	1.6 (1.4, 1.9)	1.6 (1.4, 1.9)	1.6 (1.4, 1.9)	1.5 (1.3, 1.8)	<0.001	<0.001
Serum Uric acid (μmol/L)	270.0 (237.0, 314.8)	296.0 (252.0)	322.0 (278.0, 375.0)	358.0 (300.8, 419.3)	<0.001	<0.001

WC: waist circumference, BMI: body mass index, SBP: systolic blood pressure, DBP: diastolic blood pressure, FBG: fast blood glucose, TG: triglyceride, TC: total cholesterol, LDL: low-density lipoprotein, HDL: high-density lipoprotein.

**Table 2:** Prevalence of metabolic disease conditions across serum vitamin A quartiles in children and adolescents.

Metabolic Condition	Serum Vitamin A Levels				*p*-Value	*p* for Trend
	Q1	Q2	Q3	Q4		
Weight groups					<0.001	<0.001
Others	627 (82.9)	587 (77.6)	552 (72.4)	474 (63.4)		
Overweight	71 (9.4)	103 (13.6)	114 (15.0)	134 (17.9)		
Obesity	58 (7.7)	66 (8.7)	96 (12.6)	140 (18.7)		
Metabolic syndrome					<0.001	<0.001
No	740 (97.9)	733 (96.8)	726 (95.3)	671 (89.5)		
Yes	16 (2.1)	24 (3.2)	36 (4.7)	79 (10.5)		
Obesity phenotype					<0.001	<0.001
MHNO	691 (91.4)	679 (89.7)	651 (85.4)	586 (78.1)		
MHO	49 (6.5)	54 (7.1)	75 (9.8)	85 (11.3)		
MNHNO	7 (0.9)	12 (1.6)	15 (2.0)	24 (3.2)		
MNHO	9 (1.2)	12 (1.6)	21 (2.8)	55 (7.3)		

MHNO: metabolically healthy non-obese, MHO: obese but absence of MetS, MNHNO: non-obese subjects with MetS, MNHO: obese subjects with MetS.

**Table 3:** Association of metabolic risk factors and serum vitamin A in logistic regression analysis in 7–17 years males and females.

Metabolic Risk Factors	Vitamin A Quantiles				*p* for Trend
	Q1	Q2	Q3	Q4	
Metabolic syndrome					
Model 1	1 (reference)	1.514 (0.798–2.874)	2.293 (1.261–4.169)	5.445 (3.150–9.413)	<0.001
Model 2	1 (reference)	1.479 (0.777–2.813)	2.244 (1.221–4.123)	5.257 (2.968–9.310)	<0.001
Central obesity					
Model 1	1 (reference)	1.404 (1.037–1.899)	1.961 (1.469–2.617)	3.299 (2.503–4.349)	<0.001
Model 2	1 (reference)	1.444 (1.063–1.962)	2.257 (1.674–3.042)	4.135 (3.073–5.564)	<0.001
Elevated blood pressure					
Model 1	1 (reference)	1.065 (0.868–1.307)	1.014 (0.826–1.245)	1.059 (0.863–1.300)	0.260
Model 2	1 (reference)	1.150 (0.934–1.416)	1.224 (0.989–1.515)	1.420 (1.114–1.770)	<0.001
Elevated FBG					
Model 1	1 (reference)	0.916 (0.515–1.629)	1.282 (0.752–2.184)	1.092 (0.628–1.900)	0.733
Model 2	1 (reference)	0.835 (0.467–1.491)	1.075 (0.619–1.868)	0.820 (0.456–1.475)	0.386
Low HDL					
Model 1	1 (reference)	0.637 (0.384–1.054)	0.632 (0.382–1.047)	0.798 (0.496–1.284)	0.516
Model 2	1 (reference)	0.607 (0.364–1.013)	0.549 (0.325–0.927)	0.611 (0.366–1.018)	0.121
High TG					
Model 1	1 (reference)	1.469 (1.030–2.096)	2.003 (1.428–2.811)	4.706 (3.439–6.441)	<0.001
Model 2	1 (reference)	1.515 (1.060–2.166)	2.072 (1.466–2.930)	4.903 (3.524–6.820)	<0.001
General Obesity					
Model 1	1 (reference)	1.149 (0.795–1.661)	1.735 (1.231–2.444)	2.762 (1.996–3.822)	<0.001
Model 2	1 (reference)	1.227 (0.843–1.784)	2.277 (1.592–3.256)	4.101(2.877–5.845)	<0.001
High LDL					
Model 1	1 (reference)	1.837 (1.054–3.204)	1.825(1.046–3.182)	2.798 (1.656–4.729)	<0.001
Model 2	1 (reference)	1.940 (1.109–3.394)	2.156(1.223–3.799)	3.779 (2.177–6.559)	<0.001
High TC					
Model 1	1 (reference)	1.868 (1.190–2.933)	1.963 (1.255–3.069)	2.753 (1.794–4.226)	<0.001
Model 2	1 (reference)	1.910 (1.213–3.008)	2.125 (1.347–3.355)	3.318 (2.115–5.205)	<0.001
Hyperuricemia					
Model 1	1 (reference)	1.750 (1.330–2.304)	3.338 (2.575–4.326)	6.816 (5.280–8.797)	<0.001
Model 2	1 (reference)	1.565 (1.167–2.099)	2.641 (1.988–3.507)	4.709 (3.552–6.242)	<0.001

Model 1 not adjusted; Model 2 adjusted by age, gender, area, screen time, physical activity time. FBG: fast blood glucose, HDL: high-density lipoprotein, TG: triglyceride, LDL: low-density lipoprotein, TC: total cholesterol.

## Data Availability

The datasets used and/or analyzed during the current study are available from the corresponding author on reasonable request.

## Supplementary Materials

The following supporting information can be downloaded at: https://www.mdpi.com/article/10.3390/nu14030610/s1, Supplementary Table S1: Association of metabolic risk factors and serum vitamin A in Logistic regression analysis in 7–17 years males; Supplementary Table S2: Association of metabolic risk factors and serum vitamin A in Logistic regression analysis in 7–17 years females.
