# Supplementary material for: Associations between Serum Vitamin A and Metabolic Risk Factors among Eastern Chinese Children and Adolescents
Source: Nutrients. 2022 Jan 30;14(3):610. doi: 10.3390/nu14030610 (PMC8839095; doi:10.3390/nu14030610)
Supplement: Supplementary file 1 [file nutrients-14-00610-s001.zip › nutrients-1540531-supplementary.pdf]

Supplementary Table S1. Association of metabolic risk factors and serum vitamin A in Logistic regression analysis in 7-17 years males

Cardiovascular risk factors	Vitamin A quantiles				*p* for trend
	Q1	Q2	Q3	Q4	
Metabolic syndrome					
Model 1	1(reference)	0.984(0.405-2.392)	2.238(1.033-4.849)	5.984(2.997-11.951)	＜0.001
Model 2	1(reference)	0.939(0.385-2.288)	2.014(0.912-4.448)	5.321(2.560-11.060)	＜0.001
Central obesity					
Model 1	1(reference)	1.407(0.938-2.112)	2.124(1.434-3.147)	4.014(2.771-5.807)	＜0.001
Model 2	1(reference)	1.446(0.959-2.181)	2.482(1.648-3.738)	5.357(3.569-8.039)	＜0.001
Elevated blood pressure					
Model 1	1(reference)	1.179(0.886-1.570)	1.069(0.797-1.435)	1.199(0.900-1.598)	0.332
Model 2	1(reference)	1.285(0.961-1.720)	1.370(1.005-1.869)	1.736(1.266-2.382)	0.001
Elevated FBG					
Model 1	1(reference)	0.876(0.448-1.711)	1.396(0.751-2.595)	0.727(0.359-1.472)	0.741
Model 2	1(reference)	0.803(0.409-1.579)	1.058(0.552-2.028)	0.507(0.239-1.076)	0.152
Low HDL					
Model 1	1(reference)	0..476(0.235-0.967)	0.526(0.259-1.069)	1.000(0.559-1.794)	0.927
Model 2	1(reference)	0.444(0.216-0.912)	0.389(0.184-0.820)	0.621(0.324-1.190)	0.184
High TG					
Model 1	1(reference)	1.200(0.723-1.991)	1.717(1.056-2.793)	4.434(2.871-6.850)	＜0.001
Model 2	1(reference)	1.253(0.752-2.086)	1.836(1.111-3.033)	4.868(2.935-7.480)	＜0.001
General Obesity					
Model 1	1(reference)	1.037(0.659-1.631)	1.809(1.185-2.762)	2.863(1.926-4.257)	＜0.001
Model 2	1(reference)	1.112(0.704-1.758)	2.346(1.510-3.647)	4.336(2.801-6.713)	＜0.001
High LDL					
Model 1	1(reference)	1.810(0.824-3.972)	1.654(0.733-3.731)	2.716(1.291-5.712)	0.012
Model 2	1(reference)	0.915(0.868-4.224)	2.083(0.904-4.800)	4.024(1.813-8.931)	0.001
High TC					

Model 1	1(reference)	1.575(0.827-2.999)	1.232(0.618-2.454)	2.234(1.211-4.119)	0.020
Model 2	1(reference)	1.675(0.874-3.211)	1.600(0.786-3.257)	3.500(1.800-6.807)	＜0.001
Hyperuricemia					
Model 1	1(reference)	1.797(1.282-2.519)	4.101(2.945-5.710)	10.076(7.198-14.106)	＜0.001
Model 2	1(reference)	1.583(1.086-2.305)	2.426(1.667-3.530)	4.924(3.376-7.181)	＜0.001

Model 1 not adjusted; Model 2 adjusted by age, area, screen time, physical activity time. FBG: fast blood glucose, HDL: high-density lipoprotein, TG: triglyceride, LDL: low-density lipoprotein, TC: total cholesterol.

Supplementary Table S2. Association of metabolic risk factors and serum vitamin A in Logistic regression analysis in 7-17 years females

Cardiovascular risk factors	Vitamin A quantiles				*p* for trend
	Q1	Q2	Q3	Q4	
Metabolic syndrome					
Model 1	1(reference)	2.420(0.920-6.367)	2.489(0.963-6.430)	4.667(1.897-11.478)	＜0.001
Model 2	1(reference)	2.488(0.941-6.576)	2.598(0.995-6.781)	5.022(1.991-12.671)	＜0.001
Central obesity					
Model 1	1(reference)	1.396(0.887-2.197)	1.843(1.202-2.825)	2.576(1.693-3.920)	＜0.001
Model 2	1(reference)	1.451(0.918-2.293)	2.009(1.300-3.106)	3.041(1.959-4.720)	＜0.001
Elevated blood pressure					
Model 1	1(reference)	0.957(0.713-1.283)	0.959(0.721-1.277)	0.929(0.692-1.247)	0.646
Model 2	1(reference)	1.029(0.763-1.387)	1.100(0.820-1.477)	1.167(0.856-1.590)	0.290
Elevated FBG					
Model 1	1(reference)	1.014(0.324-3.173)	1.219(0.419-3.548)	2.247(0.845-2.977)	0.077
Model 2	1(reference)	0.912(0.290-2.873)	1.057(0.358-3.122)	1.792(0.651-4.930)	0.195
Low HDL					
Model 1	1(reference)	0.882(0.424-1.835)	0.790(0.380-1.642)	0.497(0.210-1.177)	0.116
Model 2	1(reference)	0.865(0.412-1.816)	0.770(0.364-1.630)	0.469(0.192-1.144)	0.101
High TG					
Model 1	1(reference)	1.786(1.083-2.948)	2.290(1.422-3.686)	5.013(3.186-7.889)	＜0.001
Model 2	1(reference)	1.825(1.102-3.022)	2.357(1.453-3.823)	5.195(3.246-8.314)	＜0.001
General Obesity					
Model 1	1(reference)	1.396(0.733-2.660)	1.837(1.005-3.357)	2.708(1.511-4.853)	＜0.001
Model 2	1(reference)	1.493(0.779-2.862)	2.133(1.151-3.943)	3.663(1.986-6.756)	＜0.001
High LDL					
Model 1	1(reference)	1.867(0.850-4.102)	1.964(0.912-4.227)	2.884(1.375-6.049)	0.005
Model 2	1(reference)	1.968(0.892-4.339)	2.228(1.025-4.844)	3.604(1.673-7.763)	0.001
High TC					

Model 1	1(reference)	2.197(1.165-4.142)	2.658(1.446-4.889)	3.339(1.826-6.105)	＜0.001
Model 2	1(reference)	2.183(1.154-4.129)	2.695(1.455-4.993)	3.460(1.858-6.443)	＜0.001
Hyperuricemia					
Model 1	1(reference)	1.718(1.046-2.820)	3.186(2.024-5.017)	5.429(3.479-8.471)	＜0.001
Model 2	1(reference)	1.599(0.970-2.637)	2.760(1.741-4.375)	4.353(2.758-6.869)	＜0.001

Model 1 not adjusted; Model 2 adjusted by age, area, screen time, physical activity time. FBG: fast blood glucose, HDL: high-density lipoprotein, TG: triglyceride, LDL: low-density lipoprotein, TC: total cholesterol.
